# Book Reviews

**Published:** 1811-10

**Authors:** 


### CRITICAL ANALYSIS

OF

### RECENT PUBLICATIONS

IN THE

DIFFERENT BRANCHES OF PHYSIC, SURGERY, AND MEDICAL PHILOSOPHY.

---

*De la Methode Jatraleptique, ou Observations Pratiques sur l'efficacite des Remèdes administrés par la voie de l'absorption cutanée dans le traitement de plusieurs Malades internes et externes ;*

*Et sur un nouveau Remède dans le traitement des Maladies veneriennes et lymphatiques ; par* J. A. Chrestien, Docteur en Médicine de l'Université de Montpellier, ancien Medicin de l'Hopital Militaire sedentaire, Medicin du Lycée de la meme Ville, et Membre de plusieurs Sociétés academiques del'Empire, et etrangéres. 8vo. pp. 464. Paris. 1811.

We have inserted this gentleman's titles at length, though we doubt that even their authority will not secure him from being ranked amongst the Charlatans whose assertions astonish the weak and convince the credulous alone. We must however admit that he has opened an extensive field of inquiry, and cannot be justly condemned till we have gone over the same ground and proved his experiments to be false. The mode of introducing medicinal substances into the system by means of friction has been explained, and many facts in support of its utility and efficacy have been stated from time to time in this Journal, by Mr. Ward, who has lately republished them in a separate volume with additional matter. We are, therefore, prepared to admit many facts related by Dr. Chrestien, which we might otherwise have disputed. Certainly he has pushed the practice much further, and appears to have derived much more success from it, than any author whom we have yet read on the subject. From the introduction we learn that this is the third impression of his *Observations sur l'emploi des remèdes à l'exterieur* ; and that he has each time altered the title of his book ; the first, *Methode par Absorption*, was improper, because the action of absorption is independent of friction. In some instances the skin absorbs the medicament rubbed upon it ; in others the remedy acts by sympathy ; or the effect may be produced by the friction merely. The second edition appeared with the title of Methode Jatroliptice, but an erudite friend, persuaded the author this was an an improper term; he has, therefore, substituted Jatraleptique for Jatroliptice, which he had found in James's dictionary, and which is certainly as expressive as the epithet he has now adopted.

> "Strange that such difference should be
> Betwixt a little c and t!"

It seems as impossible for a Frenchman to write without boasting, as to harrangue without grimace; Dr. Chrestien is sufficiently diffident of his pretensions to science, but extremely eager to convince us that his probity is unquestionable. "Si je jouis de quelque consideration, (says he) comme Medicin practicien, j'en jouis encore plus comme homme probe, delicat et désinteressé; et si le degré de science dependait de moi comme la probité, la delicatesse et le desinteressement, je ne cederais en savoir à aucun Medicin connu." This, from a native of any other country, would have caused us to suspect that the author might possess any quality than that which he so much vaunted; but in a French writer it is mere usage, and we are as willing to believe the Doctor, as we were previous to his intimation, that he is the most *delicate*, *disinterested*, and *veracious* writer in existence.

The author commences the detail of his cases and experiments with Camphor; which he directed to be mixed with saliva, and rubbed in on the inner part of the thighs in doses of eight or twelve grains. The first case was an irritation in the urinary passages from the action of Cantharides on the skin, produced on the Doctor's own person; who, being attacked with sciatica, caused the part affected to be rubbed with forty grains of Cantharides finely powdered and mixed with saliva, the effects of which he thus states:

> "La friction administrée par une main vigoureuse, armée d'un gant de peau de daim, fut forte et long-temps continuée. Je ressentis l'effet assez ordinaire des cantharides sur les voies urinaires. Il me fit plus souffrir que la chaleur vive que j'eprouvais sur la partie frictionnée. La nuit fut inquiete, l'operation ayant été pratiquée au moment de me mettre au lit. Le calme n'étant point retabli a l'heure de mon lever, je me frictionnai la partie interne de la cuisse avec douze grains de Camphre melés à la salive. Dans un instant l'absorption fut faite; quelques heures après, mon etat fut moins pénible. Ayant répété ma friction camphrée le soir, la nuit fut tranquille, et à mon reveil, je me trouvai dans une position contraire à cette de la veille."

Since that period the Doctor has never been disappointed in the effects of Camphor counteracting those of Cantharides on the urinary passages.

In a similar manner he administered it in cases of *erections fortes; erections fortes accompagnées de pollutions; retention d'urine; ischurie vesicale; ischurie renale; fièvre hemitritée;*

 *fièvre*

*fievre ardente compliquée de la gastro-bilieuse dypirique*; and in fevers of various descriptions, as catarrhal, remittent, bilious, &c. &c. In all of these the effects of the remedy were admirable, but we cannot explain the mode in which it operated. Dr. C. in general being content with informing us that the symptoms subsided, or the violence of the disorder abated soon after the friction.

A female, aged 36, of violent character, of a sanguine-bilious temperament, subject to frequent attacks of epilepsy, was seized *d'une delire frénétique si furieux*, that she was obliged to be tied down in bed to prevent her throwing herself upon the attendants, or leaping out of window, which she attempted more than once. Dr. Chrestien being sent for, directed twenty grains of Camphor mixed with saliva, to be rubbed on the inner part of each thigh; and to be repeated every two hours. In two hours the patient was so much better that she was unbound. When two drams of the Camphor had been used, *le calme fut parfait*. The same means were resorted to some months afterwards, and succeeded in preventing a return of the complaint with which she was threatened.

A spirituous liniment, composed of two ounces of spirit of juniper *(esprit de genievre)* half a drachm of oil of cloves *(huile de girofle)*, and as much balsam of nutmeg *(beaume de muscade)* cured diarrhœa, chorea St. Viti; and prevented abortion.

A plaster much used in Holland, (called Emplatre de *Rustaing*, from the name of the discoverer) applied to the breast, proved very efficacious in remedying or preventing the disorders arising from the want of milk, or in females who declined suckling. The author relates surprising instances of its success, indeed in his hands it never failed*; and it is now constantly employed by the Montpellier accoucheurs.

---

* Composition of the plaster

Prenez Litharge d'or, deux livres

Huile d'olives, deux livres et demie.

Cire jaune, une livre.

Terebinthine des Chio, } āā quatres onces.

Huile de laurien, }

Gomme opoponax, deux onces et demie.

Bdellium, }

Gomme ammoniaque, }

Sarcocolle, } āā deux onces.

Oliban, }

Mastich, }

Myrrh en larmes, }

Aloes succotrin, une once.

Racines d'aristoloches, deux onces.

Camphre raffiné, trois onces.—Faites, suivant l'art, un emplatre.

The utility of the external application of opium is sufficiently familiar to us. Dr. Chrestien remarks that he found very few individuals in whom opium, administered by friction, produced narcotic effects. He frequently used a combination of opium and camphor in brandy with good effect, and relates many instances of its success, in suppression of the catamenia and various other diseases.

In the application of Colocynthis by friction, in mania, we believe the author is completely original; he commences his first case, by observing, "Ennemi du mensonge et du charlatanisme, je vais rapporter les faits tels qu'ils sont."

The patient was a woman aged 40, who had been for some days subject to mania; the fits were occasionally marked by profound melancholy; occasionally by a taciturnity which nothing could disturb; and sometimes by furious delirium. Dr. Chrestien found her without fever; the bowels were costive; the skin soft, but without sensible transpiration. She refused all solid food, and took liquids with reluctance. No cause, except a catarrh with cold-shivering and a swelling on the right cheek, could be assigned for the malady. The remedies employed procured no relief. In consequence of the obstinate constipation, the author ordered sixty drops of the tincture of Colocynthis *(teinture de coloquinte)* to be rubbed on the belly; no effect was produced but an increased flow of urine. The following morning the friction was repeated, and a purgative given internally, which succeeded in opening the bowels; but the alvine evacuation was inconsiderable, and the amendment of the patient was but slight. After some days, no remedies being given, the patient refusing to drink, or to take any kind of nourishment, the bitter-apple was again repeated in substance in the dose of twenty grains, mixed with lard, and continued daily for eight days, when the maniacal state yielded; the only apparent effect produced by the frictions, appeared to be an increase of turbid urine. The frictions were continued for eight days longer, without opening the bowels or occasioning the least sensation; the patient regained her former faculties, and obtained sleep to which she had long been a stranger. The bowels remaining costive, notwithstanding the frictions, glysters were injected, and brought away "*de matieres durcies.*"

Several other cases of this sort are related; some of them by practitioners who were induced to try the remedy upon Dr. Chrestien's authority. In most of these the bowels remained constipated during the frictions, whilst the discharge of urine was augmented.

The author's experiments with Digitalis in cases of dropsy were were also very successful, and a case of ascites combined with anasarca, which had resisted the frictions with the tincture of Digitalis, was happily cured by a decoction of it thrown up the rectum by injection. We shall give an extract from this extraordinary narrative in the author's own words.

"Les frictions avec la teinture de digitale, si elles n'avaient pas été sans effet, n'avaient servi qu'à ralentir les progrès du mal, qui étoit cependant parvenu au point de faire craindre une mort prochaine. Ayant proposé à M. Méjan, médecin praticien du premier mérite de notre ville, de faire l'essai de la décoction de digitale pourprée, injectée dans l'anus, il y consentit. La décoction fut préparée avec deux gros de cette plante pour quatre onces de colature, et employée trois fois le jour, au moyen d'une petite seringue à injection qu'on ne vidoit qu'une seule fois à chaque opération. La dose de digitale fut de trois gros en renouvelant la decoction ; et enfin de demi-once. A peine la troisième dose fut-elle épuisée, qu'il survint un flux d'urine si considérable, que dans trente-six heures le malade en rendit vingt pintes. Les enflures se dissipérent, et l'epanchement parut ne plus exister.

"Quelques mois après cette heureuse crise, les mêmes phénomènes ayant reparu, l'emploi de la decoction de digitale à demi once pour quatres onces de colature, eut le même succès que la première fois."

Many pages of this volume are occupied with practical remarks upon Cinchona. The author employed the tincture of it in friction with advantage in cases of rheumatism, intermittent fever, &c. ; and suspecting from the little bark contained in the tincture, that the effect might be produced by the application of the spirit merely, he substituted a mixture of brandy and spirit of wine, but it was of no use in intermittent fever. He has detailed many cases in which he gave the resin or extract of bark internally. The chief advantage consists in a small dose being sufficient.

We now come to the wonderful wonder, or *nouveau remède anti-venerien*. The author prepares us for the golden novelty, by observing that accident has often effected the most important discoveries ; the accident which succeeded in the present instance was simple enough ; and accords with Pliny's remark, "nullum esse librum tam malum, ut non aliqua parte prodesset." Dr. Chrestien, it seems, had read many treatises upon syphilis, some of the authors of which attributed the curative effect of mercury to its weight. The Doctor did not lend implicit faith to this opinion ; but without stopping to inquire in what way mercury does act, he rather sagaciously thought, that if it were true that this mineral cures by its heaviness, gold being more ponderous, ought to be more efficacious. Consulting different writers on Materia Medica, he found them concur in stating, that as a medicament, gold was was useless or dangerous. On this account, and not exactly knowing what preparation of the metal to use, he relinquished his design for some years; when on perusing Clare's treatise on the use of calomel rubbed within the mouth, he felt a strong desire to make the experiment which he had not yet essayed.

He accordingly prepared an amalgam of gold, and volatilized the mercury, by the action of heat, and occasionally with nitric acid. The effect in either case was successful. He commenced his experiments upon paupers, and was astonished at the salutary effects of the remedy. Being utterly incapable of explaining them, he consoled himself with a wise saying of his master, M. de Lamure,—"Le vrai medecin, me disait mon oracle, n'est pas celui qui explique la maniere d'agir d'un remede, mais celui qui saisit bien les indications, qui fait les applications á propos, et qui en observe judicieusement les effets;" and with great naiveté concluded, "Je renoncai á toute explication."

Although Dr. Chrestien might be convinced that no particles of mercury remained in combination with the gold, the public might be more incredulous, especially since Fourcroy had pronounced it was impossible completely to disengage the gold from every particle of the mercury with which it had been amalgamated. Dr. C. therefore, to obviate the possibility of attributing his cures to the mercury which might still remain combined with the gold, prepared an oxide of this metal, by precipitating a solution of it in nitro-muriatic acid, with potass. He also prepared an oxide precipitated by tin; and some years afterwards, a muriate of gold.*

Of these preparations, the three first appear to be possessed of nearly equal powers, and were administered by friction on the gums, inside the mouth, &c. by friction, to the extent of three grains in the day. The muriate was more deliquescent and highly caustic. This induced Dr. C. to combine it with muriate of soda; he thus obtained a triple compound, more convenient for use, possessing the same properties, but in greater degree than the other preparations of the precious metal. The usual dose of the triple muriate of gold and soda was the fifteenth of a grain; it never could be pushed beyond a tenth without exciting fever, irritation, and inflammatory symptoms. In some cases it was found advantageous to use the muriate and the oxide at the same time.

The appearances of syphilis being familiar to most practitioners, it is unnecessary to extract any of the numerous

* We shall, on a future occasion, have an opportunity of explaining these preparations more minutely.—Ed.

cases cases recorded in the volume before us, of every form of the disease from blenorrhœa to long-standing confirmed lues. The complaint yielded to the gold in most instances sooner than under similar circumstances of severity it would have done to mercury. When the triple muriate was employed, in general, three grains sufficed. During the use of the remedy neither confinement nor alteration of regimen were necessary. Patients of either sex, and of any temperament, may employ it at all seasons of the year; and whilst under its action topical applications were seldom required, chancres healing, and worts disappearing by simply observing cleanliness. The patient is directed to retain the medicine in the mouth for one minute after friction, though no harm will accrue from swallowing it. It may be applied to any part within the mouth, taking care only to avoid the teeth. In three instances the author was obliged to have recourse to a different method; the patients were scorbutic, and blood flowed on the slightest friction. In females he observes we have "*une voie de plus, celle de la face interne des grandes levres;* j'y ai eu recours une fois seulement et avec succés, la femme qui avoit besoin d'un traitement anti-vénérien n'ayant jamais voulu consentir a porter le remède dans la bouche de peur de se la gater."

Besides this remarkable influence upon venereal affections, the remedy operates beneficially upon glandular swellings, and schirrous womb. The following case is less wonderful than many others which we might have quoted.

A woman aged twenty-two, who had borne one child, applied to Dr. Chrestien for a complaint which he considered as a serious affection of the womb. An examination being made by Messrs. Bourquenod, Laborie, and Fages, they discovered a schirrous tumour of considerable size. M. Laborie had found the womb in the same state six months before; but notwithstanding the remedies employed, at the period of the second examination, her pains had become more severe. She now began the friction of half a grain of the oxide of gold, gradually increasing it to one grain. In forty-five days M. Fages ascertained by examination that the tumour was sensibly diminished. The treatment being continued ten weeks, M. Laborie found that the happy change observed by M. Fages was confirmed. After persevering in the plan four months, the patient being free from all pain and ailment, declined to continue the remedy. Being examined by Messrs. Laborie and Fages, they found the tumour had diminished two thirds. She had used no other remedy or application than the oxide, and had no indication of uterine disease ten years after discontinuing it.

In

In concluding this hasty sketch of a work abounding in extraordinary statements, it becomes us to mention that Dr. Chrestien ranks high as a practitioner, is much consulted, and would rather suffer than gain by false assertions. Vauquelin, Duportal, and several other French physicians and chemists, have followed him in his experiments, have made similar preparations of gold, and have applied them in practice with a degree of success which merits the attention of practitioners in this country. It is true that gold has been prescribed in different forms, and has been abandoned; the aurum potabile is in the recollection of most of us; in the two last centuries a combination of gold and mercury was employed by *Lecocq*, *Eschenreiter*, and *Bassine*; *Loss*, *Rebentrost*, and others, gave it reduced to powder for the cure of syphilis; some gave it with purgatives, and others with antimony; *Lavigne*, physician to Louis XIII, made a panacea with gold, mercury, and silver, which was commended by Hoffman. But it is obvious that these were very different preparations from the chemical compounds first discovered by Dr. Chrestien, the merits of which can only be determined by experiment. The art of medicine, unquestionably, is advanced by the acquisition of facts from whatever quarter derived: let us not, however, content ourselves with merely collecting them, but rather imitate the bee, "quæ materiam ex floribus horti et agri elicit; sed tamen eam propria facultate vertit et digerit."

---

*The London Dispensatory: containing—1st. The Elements of Pharmacy; 2d. The Botanical Description, Natural History, Chemical Analysis, and Medical* (medicinal) *Properties of the Substances of the Materia Medica.—3d. The Pharmaceutical Preparations and Compositions of the Pharmacopœias of the London, Edinburgh, and Dublin Colleges of Physicians.* Illustrated with Tables, and Plates of the Pharmaceutical Apparatus. By ANTHONY TODD THOMSON, Surgeon, &c. &c. &c. 8vo. London, 1811. pp. 793. plates 5. Longman, Rees, and Co.

IT is very justly observed by the Author of this work, that an unnecessary multiplication of books is a great evil. We fully enter into his feelings, and particularly appreciate them as applying to Pharmacy. No sooner does the London College publish a Pharmacopœia, than the Pandora of Literature opens her casket of evils, and out fly translations, epitomes, manuals, conspectuses, with a long &c. of thick octavos for the student, loose sheets of nomenclature for the shop, and smart duodecimos for the ready use of the sapient prescriber.

prescriber. But wherefore all this? money, notoriety, fame and—public good, is the impulse. Yet in this motley host there is no individual, perhaps, but may confer some benefit on society. The multitude draws attention to the subject, and as this tide in the affairs of the men rolls along, some happy genius may be swept into the flood. For adding to a catalogue already, in his own opinion, too long, the author of the London Dispensatory offers an apologetical explanation. In its general plan and arrangement the London Dispensatory resembles closely the Edinburgh, by Duncan; but though Mr. Thomson has not thought it expedient to deviate from that established work, he has gone more largely into some parts, and those we think, of essential importance. "I trust," he says, "that the alterations and additions which I have introduced, particularly in the history of the Materia Medica, will give a legitimate value to the London Dispensatory; and especially turn the attention of the student towards Medical Botany, which has been so unaccountably neglected of late years, as to be almost regarded as unnecessary in the education of a physician. Indeed, although it has always been admitted that a correct knowledge of Materia Medica and Pharmacy can be obtained by those only who have a previous knowledge of Botany and Chemistry, yet neither the Dispensatories nor the systems of Materia Medica, published in this country, have described plants in a scientific manner; or noticed in their descriptions, those characteristics which botanists have fixed on as the only means by which a plant, that is not familiar to a reader of the description of it, can with certainty be known, when he wishes to possess it, or is in any doubt regarding it when it is obtained. From the want of this degree of accuracy in the descriptions of plants, many valuable remedies used by the inhabitants of one part of the world have been lost to those of another, where they are, nevertheless, indigenous; or instead of the proper plants, other species of the same genera, which possess little or no virtue, have been employed; and even plants, not in any respect medicinal but highly deleterious, have, merely from their bearing names in common or in pharmaceutical language similar to those of some medicinal plants, been used to the material injury of the diseased."

We lament that there is ground for these remarks, and that the identity of plants affording medicinal substances is so ill ascertained. It is obvious, however, that at the present as well as in past periods, the efficacy of vegetable substances was well known to men, whose habits of life, education, and station, have precluded them from scientific acquirements. The rustics of Europe, and the savages of uncivilized civilized nations, often endowed with much native sagacity, and great dexterity in the employment of particular remedies extracted from the vegetable kingdom, know the plants which afford these remedies by particular names. They are, however, unable to distinguish them by the appellations which men of science have given to them; and often the botanist does not know to what plant the popular name belongs. We have lately met with an instance of this. Three physicians of eminence, and one of them the author of several elaborate botanical works, were applied to for the scientific or systematic name of the plant called by the older English botanist, and by the people, "*Parsley break Stone.*" Neither of them were able to assign its name or place in the Species Plantarum. We must not say this arose from ignorance, but from inattention. To prevent this evil, Mr. Thomson proposes to add to the usual account of each vegetable substance the characters of the genus to which the plant belongs, as they are given by Willdenow in his excellent edition of the Species Plantarum; and also detailed descriptions of each in the language employed by modern botanical writers. To make this more effective, it would have been well if the ingenious author had ascertained and given the popular and country names of every plant in his Materia Medica; and that the synonyms, not only of men who have written books, but of the mountain herdsman, the village quack, and the roving savage, had been subjoined.

Some idea of the author's research for the compilation of this Dispensatory may be formed, by stating the resources from whence he has drawn his materials. The liberal spirit which always actuates the President of the Royal Society, opened to him the finest library on Science and Natural History in the world. The observations on the medicinal species of Cinchona, are enriched by the remarks of Zea in the *Annales de Hist. Nat.* the researches of Humboldt in the splendid *Plantæ Æquinoctiales*, and the *Hortus Berolinensis*. Martyn, Woodville, Smith, Sowerby, the Hortus Malabaricus, the Flora Peruviana, and the Flora Danica, have opened their stores. Gaertner (de Fructibus), Bergius (Materia Medica a Regno Vegetabili), Murray (Apparatus Medicaminum), Alston (Materia Medica) and the Linnean and Philosophical Transactions have not been overlooked. In the Chemical part the systems of Murray and Thomson; the Annales de Chimie, the Journal de Physique, and the Philosophical Transactions, have afforded both the facts and principles. In the present vacillation of chemical theories, the author has certainly done right to avoid enlisting himself under any sect; and on the interesting question res-

 pecting pecting the constitution of the muriatic and oxymuriatic acids, he has cautiously withheld his opinion.

The object and intention of the work, and the means to be employed in its execution being adjusted, perspicuity in detail is accomplished by the following arrangement. Three General Heads, Elements of Pharmacy—Materia Medica—Preparations and Compounds; with their subdivisions, serve to arrange and martial the specific descriptions. The first Head, *Elements of Pharmacy*, is divided into three Sections. *Sect.* 1. Attraction, under the distinct forms of aggregation and affinity. Repulsion and the powers producing it, as caloric, light, and electricity. *Sect.* 2. Constitution and combination of substances, under solids, fluids, and gases. *Sect.* 3. Pharmaceutical operations and apparatus. As a specimen of this Head we copy the observations on Electricity and Galvanism, because the subject has at this moment a peculiar interest.

"The phenomena of Electricity depend on a very subtile fluid, which is a powerful chemical agent, capable of producing immediate decompositions and new combinations. Galvanism appears to be essentially the same as Electricity, differing, however, in some degree in its effects and the mode of production. Both are to be regarded as repulsive powers.

"Electricity may be communicated to all substances: by some it is transmitted without any perceivable obstruction, but by others with much difficulty: hence bodies in their relation to Electricity are distinguished into two classes, conductors and non-conductors; and as it can be accumulated in the latter by friction and other means, these are also denominated *electrics;* while the former are denominated *non-electrics*, to indicate their inability of being excited.

"The chemical effects of Electricity seem to depend chiefly on its power of producing a sudden high temperature; and this appears to be proportioned to the resistance opposed to its transmission. It often favors chemical combinations, as that of oxygen with metals, and promotes the instantaneous chemical union of gaseous bodies. It also effects chemical decompositions, as those of water, ammonia, alchohol, and metallic oxides.

"Galvanism may be regarded as a modification of Electricity, in which the fluid is evolved during certain chemical actions. It is transmitted through those substances which are conductors of common Electricity, and with the same degree of facility and rapidity. The metals, charcoal to a certain extent, plumbago, water, saline solutions, and the greater number of liquids, are conductors; but glass, dried and baked woods, the dry animal cuticle, and dry gases are non-conductors of the galvanic fluid. As a chemical agent, Galvanism is the most powerful of all the repulsive forces, and is capable of producing decompositions which could not otherwise be effected. By its means the chemical constitution of the alkalies and the earths has been established, and their bases discovered to be substances hitherto unknown, which have have been added to the list of metals. Galvanism, like Electricity, acts as a stimulus to the living system. Its effects on the animal body are, a sensation of light when applied to the eye; a sensation of acidity on the tongue; and the excitement of strong muscular action."

Five plates of pharmaceutical apparatus, of particularly correct and spirited outline, are attached to this division of the work.

The second part, or what has, perhaps, been too confinedly called the Materia Medica, forms the most interesting division of the volume; and in this, as connected with natural history and botany, will be more particularly seen, what Mr. Thomson conceives to be an improved method of treating the subject By giving the article on the genus *Cinchona*, we shall present our readers with, what we admit to be, an advantageous specimen of the author's execution of his design.

" Cinchona, *Spec. Plant. Willd.* 1. 957.
" *Cl.* 5. *Ord* 1. Pentandria Monogynia, *Nat Ord.* Contortæ *Linn.* Rubiaceæ. *Juss.*
" G. 346. *Corolla* funnel-shaped. *Capsule* inferior, two-celled, bipartite with a parallel partition. *Flowers downy, with the stamens included.*
" *Species* 1. Cinchona *officinalis.* Officinal Cinchona. Cinchona Lancifolia. *Mutis, Zea, Annales de Hist. Nat.* tom 2. p. 207. C. Condamenia *Humboldt, Plantæ æquinoctiales,* p. 32. t. 10. *Lambert. A Description of the Genus Cinchona.* plate i.
" *Species* 3. Cinchona *macrocarpa.* Long fruited Cinchona. Cinchona cordifolia. *Mutis, Zea,* l. c. 11. 214. C. purpurea. *Flor. Peruv.* 32. t. 193. C. ovata. *Ruiz.* Quinologia. C. micrantha. *Flor. Peruv.* 52. t. 194.
" *Species* . Cinchona *oblongifolia. Mutis, Zea,* l. c. 11. 211. C. lutescens. *Flor. Peruv.* 11. 53. t. 196. and Ruiz Quinologia. art. vi. 71.
" *Corollas smooth with the stamens displayed.*
" *Species* 4. Cinchona *caribaea.* Caribean Cinchona. *Wright. Phil. Trans.* lxvii. 504 t. 10

" This important genus is not yet altogether freed from the ambiguity which has so long involved it; and although much has been effected by the industry of the Spanish botanists whom their government sent out to make inquiries concerning it, yet many species remain undescribed*, from which it is very probable the bark-gatherers collect some part of the large cargoes which are annually sent to Europe†. The three kinds

* In a large colleetion of dried specimens of the genus Cinchona in my possession, which were collected in 1805, both near Loxa and Santa Fé, I find several species which are not mentioned in the works of any of the Spanish botanists.

† Humboldt informs us that the quantity of cinchona bark annually exported from America is 12,000 or 14,000 quintals. The kingdom of Santa Fé furnishes 2.000 of these, which are sent from Carthagena: 110 are furnished by Loxa; and the provinces of Huamanga, Cuenca, and Jaen de Bracamorros, with the thick forests of Guacabamba and Ayavaca, furnish the rest, which is shipped medicinally used have been distinguished and named by Mutis, a celebrated botanist who has resided in the neighbourhood of Santa Fé de Bogota since 1783, as director of the exportation of bark; and his observations have been fully detailed by his pupil Zea; whilst the travels of Humboldt and Bonpland have afforded them an opportunity of ascertaining accurately, and describing, the species first delineated by Condamine, and named by Linnæus *officinalis*, under which term no less than four distinct species were confounded by that distinguished naturalist in the different editions of his system*. Under this trivial name also the British pharmacopœias placed as varieties the three barks known in the shops; and this error is still retained by the Edinburgh and Dublin Colleges; but in the last edition of its Pharmacopœia the London College adopted the trivial names of Mutis and Zea; and although, for the sake of uniformity, we have set down the two first species above, as named and arranged by Willdenow, yet we shall describe them under the corrected appellations.

1. Cinchona cordifolia. *Mutis.*

*Officinal.* Cinchonæ cordifoliæ cortex. *Lond.* Cinchonæ officinalis cortex, a. *communis.* Edin. Cortex peruvianus, *Dub.* Heart-leaved Cinchona: (The common pale Bark of the shops.)

The tree which affords this bark is found in the mountains of Quito and of Santa Fé, growing along their skirts, and on the plains; flowering from May to September. It is a spreading tree, rising on a single, erect, round stem of no great thickness; and covered with a smooth bark externally of a brownish grey colour. The younger branches are quadrangular, smooth, leafy, sulcated and tomentose: the leaves, which are about nine inches in length, are opposite, petiolate, spreading, of an oblong-oval, cordate or egg-shape, entire, shining on the upper surface, ribbed and pubescent on the under; with the petioles flat on one side and roundish on the other, about a thumb's breadth in length, and of a purple colour. The flowers appear in large, terminal, leafy panicles, supported on long compressed tetragonous peduncles. The calyx is five-toothed, downy, and of a purple colour: the corolla internally tomentose; the tube of a diluted red colour; the limb shaggy, white above and purplish below; and the segments spreading, with reflecting tips. The filaments are short, supporting linear anthers, bifid at the base. The germen is tomentose, and changes to an oblong, narrow capsule, about one inch and a half in length, marked with ten striæ, of a reddish brown colour, and crowned with the calyx.

The bark yielded by this tree is named *Quina amarilla*† by the Spaniards.

shipped from Lima, Guayaquil, Payta, and other ports on the South Sea. *Plantæ Equinoc.* p. 34.

* Under this name in the *Sys. Veg.* ed. 10. p. 929, is described the C. *officinalis*, the *Condaminea* of Humboldt; but in ed. 12. 164, it is the C. *cordifolia* of Mutis, the *macrocarpa* of Willdenow: in the 13th ed. p. 178, it is the C. *pubescens* of Vahl, figured by Lambert, pl. 2. and lastly the C. *nitida* of Ruiz and Pavon. *Flor. Peruv.* ii. 50 *t.* 191.

† *Yellow* or *pale bark*; the adjective signifying both yellow and pale, or wan. The name appears to be used in contradistinction to *narranjada*, orange colour, which is applied to the next officinal species.

niards. Two other varieties of it, probably produced by distinct species, are also known in commerce by the names of *lagartado* (lizard like) and *negrillo* (blackish), from the colour of their epidermis. It has always been known in this country by the vague name of Peruvian or officinal bark; and erroneously regarded as the kind produced by the tree which was delineated by Condamine. It is decorticated in the dry season, from September to November, which is the period at which all the kinds are barked, and the bark is carefully dried in the sun. The trees generally die after the operation.

The bark arrives in Europe packed in chests made of slips of wood roughly fastened together, and covered with skins; each of which contains about 200 lbs. weight, well packed, but generally containing a quantity of dust and other heterogeneous matter. It consists of pieces eight or ten inches in length, some of them scarcely one-tenth of an inch in thickness; singly and doubly quilled, or rolled inward, the quills being scarcely larger than a swan's quill*; and others of a coarser texture, thicker and nearly flat. Both kinds have a chopped, greyish or cineritious epidermis, often covered with flat, sometimes stringy lichens; and internally of a cinnamon hue. They are evidently the bark of the same tree; the quilled sort being that of the smaller branches, and the flat that of the larger and the trunk. But the chests probably contain similar barks obtained from different species.

*Qualities.* Good bark of this description has scarcely any odour when in substance; but during decoction the odour is sensible, and agreeably aromatic. The taste is bitter, but not unpleasant, slightly acidulous and austere, resembling in some degree that of a dried rose. It is light, and breaks with a close fracture, with the internal fibres somewhat drawn out. The powder is paler than the bark, of a fawn colour, or a light cinnamon hue; but the flat kind yields a deeper coloured and browner powder. The best specimen of this bark which could be procured by me, and subjected to experiment, gave the following results: Water at 160° extracted all its active principles; affording an infusion, when filtered, of a pale yellow or straw colour, which had the odour and taste of the bark. It reddened litmus paper; was instantly and copiously precipitated by solution of galls; and in a smaller degree, and more slowly, in yellowish flocculent flakes, by solution of isinglass. A solution of tartar emetic was rendered turbid, and slowly precipitated by it; but this effect was quickly and copiously produced on superacetate of lead. Sulphate of iron changed its colour to bright olive-green, but was not precipitated. The powder macerated in sulphuric ether afforded a golden yellow tincture, which reddened litmus paper, and left a pellicle of bitter resin when evaporated on the surface of water, to which it gave the colour of the tincture. This coloured water had the flavour of the watery infusion, but differed from it, in not precipitating the solution of galls and tartar emetic; and in throwing down a copious precipitate from the solution of sulphate of iron. With alcohol the pow-

---

* The great desire of our bark merchants to procure quill bark has occasioned the bark gatherers often to produce this effect by heat, which never fails to diminish the virtue of the bark. *MS. of Don Felix Devoti of Lima* in the author's possession.

der der afforded a tincture of a deep orange hue, which precipitated sulphate of iron, tartarized antimony, and tannin; became turbid when added to water, and let fall a light reddish precipitate. From the effects of these re-agents on the aqueous infusion of this bark, it appears to be the same as the 3d and 15th species examined by Vauquelin; which he names *superior gray cinchona*, and *common cinchona of Peru**.

According to Mutis and Zea, it is indirectly febrifuge only; but when genuine both the varieties of it are excellent remedies.

"2. CINCHONA LANCIFOLIA. *Mutis.*

"*Officinal.* CINCHONÆ LANCIFOLIÆ CORTEX. *Lond.* CINCHONA OFFICINALIS, CORTEX, b. *flavus. Edin.* CORTEX PERUVIANUS. *Dub.* Lance-leaved Cinchona. (The yellow Bark of the shops.)

"This tree is found on the Andes of Peru, near Ayavacam, growing at heights from 6,250 to 8,300 feet, where the mean temperature varies between 59 and 62 degrees, on a bottom of micaceous schist in the woods of Caxanuma, Uritucinga, Villonaco, and Monge*. It is a lofty handsome tree, always in leaf; and exudes, wherever it is wounded, a yellow astringent juice. The trunk is about eighteen feet in height and fifteen inches in diameter, erect, with a cracked ash-coloured bark; the branches are round, in opposite pairs, erect, brachiated; with the younger ones obscurely quadrangular at the nodes. The leaves are of a lively green, shining, oval, lanceolate, about three inches long, with a little pit in the axillas of the nerves on the under surface, which is filled with an astringent aqueous fluid, and having the orifice shut with hairs; and they stand on footstalks one-sixth of their length, flat above, and convex below. The stipules are two, acute, silky, contiguous and caducous. The flowers, which are odorous, of a whitish rose colour, and furnished with little bractes, appear in terminal, brachiated, leafy, trichotomous panicles, supported on round peduncles and pedicles, that are powdered and silky. The calyx is of a globular bell shape, five-toothed, powdered and silky like the peduncles, with the teeth acute, very short, and contiguous. The corolla is somewhat salver-shaped, six times longer than the calyx, with the tube obscurely pentagonous, silky, more frequently of a rose colour; the *limb* wheel-shaped, with oval segments, much shorter than the *tube*, white and woolly above. The germen is globular, changing to an ovate, woody, longitudinally striated capsule, crowned with the calycinal teeth, two-celled, many-seeded, oppositely twice furrowed, and opening from the base to the apex with two valves.

"This tree affords the original cinchona of Peru, which is now very rare, 110 quintals only being cut, instead of 4000 which was the quantity in 1779, and reserved for the use of the Spanish government. Zea says it is a variety of the *lancifolia* of Mutis, under which we have placed it; and there is also a great affinity between it and the *scrobiculata* of Humboldt, according to that celebrated traveller. The bark of the *lancifolia* is the yellow bark of the shops, the Quina Naranjada of

* *Annales de Chimie*, lix. 116.

* As the *Condaminea* of Humboldt is evidently a variety, if not the same species meant by Mutis, we have availed ourselves of his accurate description.

the the Spaniards. It is known in commerce by the name of Calisaya*; and is preferred in South America to the pale cinchona. It is brought to this country in chests containing about 90 to 100 pounds each; and consists of pieces about eight or ten inches in length, some quilled, but the greater part flat†. The quilled pieces are less rolled and thicker than the quilled pale bark; and the epidermis, which is of a tawney grayish brown colour, and covered with flat and stringy lichens, more rough and chopped, easily separating, and often as thick as the bark itself, which is about one-eighth of an inch. The flat pieces are generally without any epidermis, and considerably thicker than the quilled: both are mixed in the same chest.

" *Qualities.* Yellow bark has nearly the same odour in decoction as the pale; the taste is more bitter, but less austere, and it does not afford any astringent feeling to the tongue when chewed. The internal colour is an orange cinnamon, or subdued yellowish brown; the fracture is woody and fibrous, presenting, when examined by a lens, the appearance of parallel longitudinal needle-like fibres, with a dry agglomerated powder in the interstices of a yellow colour. It is easily reduced to fine powder, and the powder preserves the colour of the bark, but is brighter. The filtered aqueous infusion has a pale golden hue, with a shade of red; is clearer, and seemingly less mucilaginous than the former: it has all the bitterness of the bark; reddens litmus paper, and precipitates solution of galls; but the precipitate does not fall so instantaneously as in the infusion of the former species. With solution of isinglass a pinkish yellow precipitate is produced: superacetate of lead throws down a precipitate; and that with tartarized antimony is more copious than the pale bark affords, and in yellowish white flakes. A solution of sulphate of iron changes its colour to a deeper green, and after many hours gives a precipitate of the same hue. The ethereal tincture has the same golden colour affords resin when evaporated, and is affected by the same reagents as that of the pale cinchona; but the water on which it is evaporated is less highly coloured. The alcoholic tincture appears to be in every respect the same as that afforded by the pale bark. It seems to agree in most of its properties with the first species examined by Vauquelin; which he states was brought to Spain in 1788, and, owing to its having been used for the royal family, got the name of royal cinchona.

" Mutis and Zea regard this as the only species of cinchona which is directly febrifuge; and assert that it never fails to cut short an ague when administered at its accession.‡

" 3. CINCHONA OBLONGIFOLIA. *Mutis.*

" *Officinal.* CINCHONÆ OBLONGIFOLIÆ CORTEX *Lond.* CINCHONA OFFICINALIS, CORTEX. C. *ruber*. *Edin.* CORTEX PERUVIANUS. *Dub.* Oblong-leaved Cinchona Bark. (The red Bark of the shops.)

" The tree yielding this bark is found on the Andes, growing in the

---

* The name *Calysaya* is the generic name by which the Peruvian Indians distinguish the superior barks. *MS. of Dr. Devoti.*

† These are distinguished in commerce by the terms *Calisaya with coat*, and *Calisaya without coat.*

‡ *Annales de Historia Natural*, ii. 209.

 woods woods on the banks of the mountain streams, in great abundance, at Chinchao, Cuchero, and Chacahuassi; flowering in June and July. It rises to a very considerable height on a single, erect, round stem, which is covered with smooth, brownish ash-coloured bark. The older branches are round, smooth, and of a rusty colour; the younger are obtusely four-cornered, leafy, and of a diluted reddish colour. The leaves are opposite, large, the full-sized ones being one or two feet in length, of an oblong oval shape, and supported on short semiround purple petioles. They are entire, pale, on the upper surface shining, on the under veined, with veins that turn to a purplish colour; and at the base of each are numerous bundles of white bristles: the stipules are supra-axillary, interfoliaceous, opposite, contiguous, united at the base, and of an obovate figure. The flowers appear in large, erect, much compounded terminal panicles, somewhat branched, on long brachiated many-flowered peduncles. The calyx is small, five-toothed, and of a purple colour; the corolla white and odorous, with the limb spreading, and hairy within: and the filaments are inserted into the tube of the corolla, and support oblong anthers bifid at the base. The capsules are large, oblong, obscurely striated, slightly curved, and crowned with the calyx.*

This tree is named in the vernacular Spanish *Cascarilla de flor de Azahar*, from the flowers resembling in odour those of the orange; and its bark is the *Quina roxa* and *colorada* of commerce. The bark is brought to this country in chests, which contain from 100 to 150 lbs. each. It consists of large thick pieces, covered with a thin and rough entire reddish brown epidermis. The greater number of the pieces is flat, but some are partially quilled, as if taken from half the circumference of the branches to which they belonged. Under the epidermis there is an intermediate layer, which is dark coloured, compact, brittle, and seemingly resinous; and within it the internal part is woody, fibrous, and of a rust-red colour. The fracture, examined by a lens, consists of close longitudinal parallel needle-form fibrillæ of a pale red colour, with a deep red agglomerated powder in the interstices. The powder is of a deeper colour than the internal part of the bark.

" *Qualities.* Red cinchona bark has a weak peculiar odour; and its taste is much less bitter, but more austere and nauseous, than the two former species. The aqueous infusion has a pale ruby colour, a slight degree of bitterness, and a decided astringency. It reddens litmus paper†, is slowly precipitated by the solution of galls, the supernatant liquor being perfectly colourless; and a very light, flocculent, ruby-coloured precipitate is produced by the solution of isinglass: it is not altered by tartarized antimony, nor by the seperacetate of lead; and the sulphate of iron makes it assume a dirty yellow olive colour only, without being precipitated. The ethereal tincture is of the same colour, and exhibits the same appearances as that of the two former species, when treated in a similar manner. The alcoholic is of a very deep brownish red colour; when diluted with water a red flocculent

* *Flora Peruv.* ii. 53. *t.* 196.

† Fourcroy found in it a portion of citric acid, some muriate of ammonia, and muriate of lime. See *Thomson's Chem.* v. 216.

matter matter falls down; and it precipitates the solutions of sulphate of iron, and of tartarized antimony, the former of a black colour, and the latter red. It comes nearest to the second species examined by Vauquelin, which he calls *Santa Fè cinchona*; and differs from his *Cinchona magnifolia* in reddening litmus paper, and precipitating tannin.

This bark was introduced by Don Sebastian Josef Lopez Ruiz, in 1778; and is considered by Zea and Mutis as the least directly febrifuge of the three kinds we have described.

---

" The most complete examinations of cinchona, with the view of discovering on what principle its febrifuge properties depend, have been made by Vauquelin and Fabroni. The former divides all the different species of cinchonas into three sections relative to their chemical properties*. The first comprises those which precipitate tannin, but not animal gelatine; the second, those which precipitate gelatine, but not tannin; and the third, those which precipitate at the same time tannin, gelatine, and tartar emetic. He conjectured that on the principles producing these effects, particularly that which precipitates infusion of galls, the febrifuge properties of the barks depend, and that they are more or less remarkably febrifuge, in proportion to the quantity of these principles that are present. He asserts that the principle which precipitates tannin is of a brown colour and bitter taste; is less soluble in water than in alcohol; and it also precipitates tartarized antimony, but not glue†. It has some analogies with the resinous bodies, although it furnishes ammonia on distillation: whilst the principle which in some cinchonas precipitates glue has a bitter and astringent taste; is more soluble in water than the principle, which in other kinds precipitates tan; and that it is also soluble in alcohol, and does not precipitate tartar emetic‡. Fabroni conceives that he is authorized to conclude from his experiments, that " the febrifuge virtue does not belong essentially and individually to the astringent, the bitter, or any other soluble principle, as the quantity of these increases by long boiling, while the virtues of the decoction decrease. Neither does the febrifuge virtue reside in that principle which destroys the emetic property of tartarized antimony, or precipitates iron, since the decoction contains more of it than the infusion, while its virtues are evidently less§." Hence we may conclude, from these doubts and many others that have been raised, that much is yet to be done before the effective principle of cinchonas in the cure of fevers be ascertained‖. We may, however, venture to state the

---

* He examined 17 different kinds, but was not able to ascertain the names of the trees from which they were obtained.

† The effect of this principle was first noticed by Dr. Maton; and soon after by Seguin, who immediately concluded that it was gelatine; but this opinion was proved to be erroneous by Dr. Duncan, jun. who found that it was a principle sui generis, and named it *cinchonin*. Vide *Nicholson's Journal*, vii. 226.

‡ *Annales de Chimie*, l. c. § *Edinburgh Medical and Surgical Review*, ii. 338.

‖ In consequence of a chemical theory of the mode in which cinchona acts on the living body, Fabroni made some curious experiments to ascertain the relative affinity of different cinchonas to oxygen. In imitating his experiments with the three officinal species, we found that when half a drachm of each of these barks in powder was separately mixed with half a fluid ounce of strong nitric acid, in similar vessels, the temperature of the atmosphere at the following as the known active constituents of cinchonas: *cinchonin, resin, extractive, gluten* or *ferment, volatile oil**, and *tannin*. I separated the *resin* in a pure state by evaporating the ethereal tincture on the surface of cold water; and the *gluten* Fabroni found was separable by water, occasioning the spontaneous fermentation of the decoction and infusion in summer, and decomposable by fermentation. They also contain several salts having lime for their basis, one of which, peculiar to yellow bark, Descamps, an apothecary at Lyons, discovered, and erroneously ascribed to it the febrifuge property of the bark. Vauquelin found it to consist of lime, and a peculiar hitherto unknown acid, which he denominated *kinic*, and therefore termed the salt a kinate of lime†.

"As cinchona bark occasionally varies in its powers, and is often adulterated with other inferior barks even by those who gather it; arising either from ignorance, or from a fraudulent desire of more quickly completing their contracts; it is of much importance to be able to distinguish good bark, and the best varieties from those of an inferior description. The following directions for choosing bark are those generally attended to in South America‡: The essential characteristics are *colour, taste*, and *smell*; the secondary or accidental ones are *exterior coat, fracture, weight, thickness*, and *quill*. The best bark of the first class is of an orange yellow colour; and the goodness decreases as the colour varies from this to a very pale yellow. When of a dark colour between red and yellow it is always to be rejected; as this colour designates either that it is of a bad species, or that it has not been well preserved from the air and moisture, which always diminish its virtues. This dark colour, however, must not be confounded with a red colour in the inside which constitutes a distinct species. The *taste* of bark should be bitter, but not nauseous nor very astringent, with a slight agreeable acidity just perceptible to the palate; and when chewed it should not appear in threads, nor of much length. The *odour* of any of the barks is not very strong; but when they have been well cured and preserved, it is always perceptible; and the stronger it is, provided it be pleasant, the better may the bark be considered. The appearance of the *coat* or epidermis has led to many mistakes. It is merely accidental; depending on the variation of the ground, and the exposure of the branches to the sun and air. Seven distinct appearances of the epi-

---

time being 70°, and that of the acid 71°, in the space of four minutes the heat produced rose the mercury in the thermometer as follows:

| | | |
|---|---|---|
| Common pale bark, | — to | 120° |
| ———— yellow bark, | — | 123° |
| ———— red bark, | — | 119° |

The mixture was gradually swollen as the heat increased, and nitrous fumes were given out, showing the evident decomposition of the acid.

* Dr. Irwin first obtained a small portion of this oil.

† *Annales de Chimie*, lix. l. c. The name of the acid is derived from *kina kina*, an old appellation of the bark. Dr. Duncan proposes to call it *cinchonic acid*, as the present name would lead to the supposition that it is procured from kino.

‡ Extracted from a MS. in the author's possession, of *Don Felix Devoti*, a respectable physician at Lima, who has practised upwards of twenty-five years in South America.

dermis dermis are remarked: 1. Negrillo, dark silver coat; 2. Crespillo, short curled; 3. Pardo-obscuro, dark open leopard gray; Pardo-clara, light open gray; 5. Lagartado. fine dark silver, lizard-like; 6. Blanquissimo. very pale; and 7. Ceniciento, ash coloured. The three first are the best, and belong to bark produced on the highest mountains: the others rank in the order of their arrangement; the epidermis being always cracked and rough in proportion as the trees have been exposed to a scorching sun. With regard to *fracture*, some of the worst barks break even and clean as if cut with a knife, and some of the best have alway a more or less splintery fracture*. The fibres of the fracture being sharp and short indicate the bark to have been gathered from mature branches; the long and threadlike from immature branches. The best barks are generally observed to be the heaviest. In point of *thickness*, very thin bark is deficient in strength, owing to the branches from which it was taken having been too young; and very thick bark, particularly if it breaks like common wood, argues that the tree must have been sickly: yet bark exceeding a line in thickness may be good; for although it is disapproved of at Cadiz, under the name of quinon, yet excellent effects have resulted from much thicker bark in England. The moderately thick and firm bark is always preferred at Lima. The moderate *quill* of bark certainly denotes it to be of the best kind, and that it has been taken from branches of a proper age. and well dried; but the bark collectors often produce this effect by fire, when there is a want of sun, as is frequently the case in some parts of the mountains. The fraud is known by the colour being much darker; and, when the bark is split, the inside exhibiting stripes of a whitish sickly hue.

" *Medical properties and uses.* Cinchona bark is a powerful and permanent tonic, possessing also antispasmodic and antiseptic powers; and is undoubtedly superior to all other remedies in counteracting febrile action, and restoring strength and vigour to morbidly weakened states.

" The stories which are related regarding the discovery of its febrifuge effects appear to be founded on fiction, and are unworthy of notice; but it is probable that the Peruvians were acquainted with its powers before the conquest of their country by the Spaniards, and from them the knowledge of it must have been acquired by their conquerors. It was, nevertheless, little known by Europeans, until the countess of Cinchon, wife of a viceroy of Peru, was cured by it at Lima of a tertian ague, in 1638; after which its fame beginning to spread, it was taken to Italy in 1649, and through the means of cardinal De Lugo and the Jesuits was distributed over the continent†. It was in repute in England in 1658; but owing to its high price‡, or some other cause, it was very little used, till Talbor, an Englishman, again brought it

---

* The idea of a resinous fracture being the characteristic of good bark originated when its virtue was supposed to depend on the resin it contained.

† Morton gives the above account on the authority of Bollus, a Genoese merchant, who had lived long in Peru, " autor fide dignus." *De Febribus Intermit* c. vii.

‡ It was sold at first by the Jesuits for its weight in silver; and Condamine relates that, in 1690, several thousand pounds of it lay at Piura and Payta for want of a purchaser. *Mémoires Acad. Roy.* 1738.

into into vogue by the many cures he performed with it in France, under the name of the *English remedy*, and his secret of preparing and exhibiting it was purchased by Louis XIV. and made public. Hence the origin of some of the appellations it has had: as *Cortex* and *Pulvis Comitissæ; Cortex* and *Pulvis de Lugo; Jesuit's bark;* also on account of its effects, *Palos de calentura*, or fever wood; and, from the place whence it was first brought, *Peruvian bark*.

" It was introduced into practice for the cure of intermittent fever, and still retains the reputation it acquired as a remedy for that disease; although, owing to peculiar idiosyncrasies and other accidental causes, it has occasionally failed in this country in agues which were afterwards removed by other remedies, particularly arsenic. Some of these failures may perhaps have arisen from the kind of the bark employed: for notwithstanding the generally received opinion, that all the kinds of bark may be indifferently used, one for another, yet there is some reason for the assertions of the Spanish and American physicians, that they vary in other respects besides their degree of activity. By them the yellow bark, *calisaya*, *quina naranjada**, is considered as directly febrifuge, and the best adapted for the cure of ague: the pale bark, *quina amarilla*, as only indirectly so, and better fitted for slow fevers and chronic debilities: while the red, *corolada*, *quina roxa*, is only fit to be used in cases of gangrene†, as its use is apt to be followed with disgustful nausea, severe vomiting, and insupportable colic. The differences of opinion with regard to the best time of giving it are now nearly settled. Boerhaave‡ and others recommended that the fever should be allowed to run on for some time before it was administered; but it is now generally agreed that the bark cannot be given too early after the stomach and bowels are cleared by an emetic and cathartic. Dr. Cullen recommended the exhibition of it in a large dose or doses immediately before the accessions§; but Morton's method of giving it directly after the hot stage of the paroxysm ceases, and repeating it in increased doses during the intermission, until the cold stage again returns, is now generally adopted. It may be safely given during the paroxysm, as practised by Dr. Clarke of Newcastle, but many stomachs are apt to nauseate it at that time.

" In remittent fevers cinchona is found equally efficacious, the bowels however requiring to be kept more open. It renders the remissions distinct, and by degrees checks altogether the febrile action. In other affections depending on a similar state of habit, as hemicrania, periodical pains, spasms, chorea, hysteria, epilepsy, passive hæmorrhagy, and in habitual frequently returning coughs, it is also found useful: but it does not prevent the continuance of those paroxysms of ague which form one of the constitutional symptoms of stricture of the urethra, and some other local affections; and which can be removed only by removing the strictures and other sources of irritation.

---

* According to Condamine, this was the bark first introduced in Europe. He says it yields by incision a yellow odorous resin; and that the Jesuits of La Paz (whence the best bark of this species is still obtained) used to gather it with care, and send it to Rome, where it was specific in agues. But the Loxa bark coming to Europe soon after, the two kinds were confounded together.

† Zea, *Anales de Hist. Nat.* l. c. Rushworth discovered the efficacy of the red bark in gangrene.

‡ *Aphorismi, &c.* 767.

§ *Mat. Med.* ii. 97.

"In continued fevers of the typhoid type, particularly when these are attended with symptoms of putridity, as in jail-fever, cynanche maligna, and scarlatina maligna, confluent small-pox, and in putrid measles, the bark must be regarded as one of the most valuable remedies. The administration of it in pure typhus has been of late years delayed till the increased excitement is presumed to be subdued, and symptoms of great debility make their appearance, or until the morbid heat be carried off, and the skin opened. Several eminent modern physicians*, however, recommend it to be given early in the disease, and persevered in; but from our own experience we are inclined to consider the former the safer practice, and believe that the best effects will be produced from the cinchona, when its use, in pure typhus, is not begun till the skin becomes moist, the tongue is in part cleaned, and the urine deposits a critical sediment. In the other febrile diseases, however, above mentioned, it should be given in as large doses as the stomach will bear, as soon as the typhoid symptoms become evident, and continued through every period of the disease.

"Cinchona was first conjectured to be useful in gout by Sydenham, and in some cases its efficacy is sufficiently evident. In acute rheumatism also, Dr. Haygarth has lately strongly recommended it to be given, after the manner of Morton, Hulse, and Fothergill, from the commencement of the disease; the stomach and bowels being previously emptied by means of antimonial preparations. In our own practice we have found it useful only after the liberal exhibition of calomel, tartarized antimony, and opium, when the pains have in some degree abated, and the pulse has become softer.

"In phthisis, bark is found beneficial when the accompanying hectic puts on more of the intermittent form than usual; when the debility is considerable, and blood is mixed in the sputa: and in several cases of pneumonia, when, after repeated large bleedings and evacuations, the pulse continued hard and thrilling, and the blood buffy, although the expectoration was free and the skin open, we have seen bark produce the happiest effects.

"In various cutaneous diseases, as lichen agrius, and lividus, and purpura†; in erysipelas, and extensive ulcerations both from common inflammation and venereal affections‡; in the termination of all acute diseases after the urgent symptoms are subdued; and in dyspepsia, chronic debility, and nervous affections, the use of cinchona is found to be of the greatest advantage.

"As a local remedy, bark is sometimes used in the form of gargle in malignant sore throat and aphthous affections; and as a wash to fœtid gangrenous sores. Powerful effects also are said to have been produced upon the system by frictions with the extract, softened by saliva or oil, upon the thighs and other parts of the body; but Denman says he found no advantage from its use as a clyster in the low state of puerperal fever, in which it has been highly extolled. It may be efficaciously administered per anum, when it cannot be taken into the stomach.

Cinchona bark is administered in a variety of forms. (See *Preparations*

* *Clarke* of Newcastle, *Heberden.*

*rations and Compositions.)* In substance it is reduced to the state of an impalpable powder; and although it loses some of its activity during the process of pulverization, yet, when it can be retained on the stomach, this is the best form of the remedy*. If it excite nausea or vomiting, or operate as a cathartic, or occasion costiveness, these inconveniences may in some degree be obviated by combining it with aromatics, opium, or a cathartic, as the circumstances direct: or some of the lighter preparations, in which its active principles are supposed to be extracted, and free from the grosser parts, may be employed. The powder is given mixed in wine or water: or, when the taste is an objection, in milk, or syrup, or a solution of extract of liquorice, which effectually cover the taste, provided the dose be taken directly after it is mixed.

The dose of the powder is from grs. v. to ʒij. or more. In intermittents the full dose is sometimes given at first; but in other diseases grs. v. x. or xv. are sufficient to commence with, the dose being repeated every two, three, or four hours, and gradually increased, until one or two ounces, in some cases be taken in twenty-four hours.

Officinal preparations. *Infusum Cinchonæ.* L. E. D. *Decoctum Cinchonæ* L. E. D. *Extractum Cinchonæ.* L. E. *Extractum Cinchonæ resinosum.* L. D. *Tinctura Cinchonæ.* L. E. D. *Tinctura Cinchonæ composita.* L. E. D. *Vinum Gentianæ compositum.* E.

### 4. Cinchonæ caribæa.

*Officinal.* ——CORTEX. *Edin.* The bark of Caribean Cinchona.

The tree which yields this bark is found in Jamaica and the Caribbees, growing near the sea-shore†. It rises twenty feet, sometimes fifty feet, in height, with a trunk of a small diameter, but very hard, tough, of a yellowish white colour in the inside, and covered with a cineritious bark. The branches are round in the lower part, but somewhat compressed above, of a brownish purple colour, and sprinkled with ash-coloured points. The leaves, which are on very short petioles, are of a rusty green colour, egg-shaped, pointed, entire, smooth, and veined; with small pointed stipules broader than they are long, and ciliated. The flowers are solitary, on axillary opposite peduncles the length of the petioles; the calyx is small and five-toothed; the corolla smooth, of a dusky yellow colour, with a slender tube nearly an inch in length, and the segments of the limb of the same length, and linear. The filaments are the length of the corolla, and consequently project considerably out of the tube. The style is as long, with a thickish undivided stygma; and the capsule oblong, smooth, of a black colour when ripe, and bivalvular.

Very little of the bark is brought to this country, so much so, that we could scarcely procure a specimen of it in the London shops. It is in pieces about eight inches long, about an eighth of an inch in thickness,

* Fabroni says, " Cinchona loses its solubility, and consequently its activity, by long exposure to the air, and by pulverization long protracted with the view of rendering it as fine as possible. From $\frac{12}{100}$ to $\frac{16}{100}$ are obtained from bruised cinchona, which in fine powder yields only $\frac{6}{100}$ or $\frac{7}{100}$ to water.

† *Wright, Phil. Trans.* lxvii. 504. *t.* 10. *Lambert's Description of the Genus Cinchona,* 24. *t.* 4.

ness, and quilled; with a brownish-gray epidermis, covered with white lichens.

*Qualities.* This bark, when chewed, has at first a sweetish taste, in some degree resembling the flavour of horse-radish; but becomes afterwards very bitter, austere, and nauseous.

*Medical Properties and Uses.* The Caribean cinchona bark is tonic, and, according to Dr. Wright, who first introduced it, may be advantageously used in all cases where Peruvian bark is indicated.

[To be continued.]

---

### *The Edinburgh Medical and Surgical Journal, No.* XXVII. *July*, 1811.

Art. I.—*Cases of Ruptured Spleen and Liver by external Injury, with Remarks thereon. By* C. Chisholm, M. D. F. R. S. &c. &c.

Fatal injuries of internal organs frequently occur, and when unaccompanied by marks of violence, the original cause of the mischief often escapes observation, and the patient is treated for some general disease. Thus, blows on the region of the liver or spleen have occasioned a series of symptoms terminating in death; the integuments not being injured, and the patient not recollecting the accident, the origin of the morbid action has not been suspected. The learned writer of the article before us, during an extensive practice in the West Indies, had several opportunities of witnessing such occurrences; and since his return to this country, has been enabled by his experience to direct an appropriate and successful treatment in cases which might have been fatal, had he not suspected the cause. He has, therefore, with the laudable view of instructing others, published the following interesting cases from his West India Journal.

" On the evening of the 27th July 1792, Joseph Morton, a gunner of the royal artillery, aged 25, of a moderate height, naturally strong constitution, but for a year before frequently harassed with dangerous attacks of remittent fever, and by trade a blacksmith, carrying on his back a bag containing eighteen gallons of dry peas, down the declivity of a hill of a moderate ascent, fell with his left side, exactly the region of the spleen, on a large stone. That evening, and all the following morning, the fall occasioned so little uneasiness, that he was able to work at the forge: but afterwards he became very feverish, and so oppressed with pain in his left side, the epigastrium, and lower extremities, as to be obliged to desist. On the evening of the 28th, he was brought to the Ordnance Hospital, when I found him much in the situation mentioned. I bled him very freely, which, however, so far from giving him relief, seemed to increase his fever to an outrageous degree of delirium. Upon this, imagining that there might be more of a nervous vous excitement than of inflammatory diathesis, I ordered him a pill of gr. i of opium, and gr. ij of James's powder. This gave him no relief. A second pill was given two hours after the first; and half an hour after taking it, he became calm, and fell into a profound sleep. On the morning of the 29th he had still much fever, but the pains were considerably abated. To remove the fever, he took saline mixture, with small portions of sweet spirit of nitre and antimonial wine. On the 30th he complained of pain in his throat, and excessive difficulty in swallowing. For these a blister was applied between the shoulders; and to obviate costiveness and some remains of fever, a solution of Glauber's salts and tartar emetic was given with desired effect. On the 31st all his symptoms had disappeared, and he felt himself apparently easy and well. About midnight, however, he suddenly became delirious with burning fever, and such an increase of pain in his left side particularly, as to render even the gentlest touch insupportable. I should have mentioned that during all the 31st he took a good deal of bark and wine, which towards evening his stomach rejected. On the 1st of August he was in this state, his skin parched and dry, and his pulse irregular, tremulous, and frequently intermitting. In addition to all this, irritability of the lower extremities excessive, with sudden spasms of the muscles; anxiety great, with grievous moaning. In pretty nearly this state he continued till one P. M. when he expired. The heat of his skin increased to an astonishing degree some time before his death; and even two hours after, when I examined the body, the heat of the abdominal cavity, and its contents, was extremely disagreeable, and of such a penetrating nature, as to leave a painful sensation on the hand some time after withdrawing it.

"I opened the body in the presence of Lieutenant Swiney of the artillery, and Mr. Campbell my assistant. I found all the intestines, the liver, kidneys, diaphragm, and heart, in a healthy state. But on the posterior superior convex side of the spleen, there was a rupture through the whole of its substance, at least two inches in length, the edges of which had a florid appearance in some places, and in others something like sphacelus could be perceived. The spleen itself was of a considerable size, but in other respects had no appearance of disease. The lungs were evidently much inflamed. All the ribs were sound, and the integuments had nowhere any mark of injury, except an almost imperceptible discoloration immediately over the spleen."

The second case was a soldier who fell from a height on the edge of a form. He complained of internal pain in the side, but no external injury could be perceived; and camphorated spirit of wine being applied, in a few days the pain ceased. No fever took place till the 11th of August, about three weeks after the accident, when the symptoms resembled those of endemic remittent fever. For this he took mercury in the following form: R. Nitri ℈j. calomel gr. iv. tart. emet. gr. ¼. camphor gr. v. M. ft. pulvis, 4 ter in die sumendus.

In the evening of the 12th, besides the four powders, he had a bolus with six grains of calomel; and on account of his his fall, was bled freely. On the 13th, complaining of much pain in his neck, back, and head, a large blister was applied between the shoulders.

" 'The powders hitherto had acted merely as deobstruents, producing most copious and frequent bilious stools. On the 14th much easier, but stomach rather irritable, for which he took saline draughts with a large proportion of the alkali. 15th.—Still better, except the irritability. Had two pills of opium of gr. i each, and swallowed two ounces of bark, but soon after vomited them. 16th.—Irritability the same; a yellow suffusion on the albugineæ of the eyes; some degree of wildness in the stare of them, but perfectly cool and moist, and pulse natural, both in fullness and quickness. A blister was applied to the stomach, with a repetition of the opium. 17th.—The blister produced a total change in the state of the stomach; so that from the 16th, on which it was applied, till the 20th, on which the patient died, he retained every thing that was given to him; but the fever returning this day, blisters were applied to the inside of the thighs, and an opening draught given to him; the yellowness still increasing. 18th.—The yellowness has gradually diffused itself over the whole surface, attended with remarkable dryness of the skin; pungent disagreeable heat; some derangement of intellect; parched mouth and tongue; black crust on the latter; saffron-coloured urine; and very fœtid frequent stools. Stomach remarkably retentive, which, under the present circumstances, is a very extraordinary symptom. Considering the disease now as a true yellow remittent fever, I gave the patient the following mixture, which on former occasions, in nearly similar circumstances, produced the best effect when used at this period of the disease. ℞. Sal. tart. ʒij. succ. limon, ℥i. pulv. rhei, ʒi. pulv. cort. Peruvian. ℥ij. aq. ℔. i . M. cyath. sumend. 3. q. q. horâ. And I continued the calomel gr. v. cum opii gr. i. also four times in the day. 19th.—The bark mixture was repeated without the lime juice on account of the mercury; symptoms continue; almost always in a comatose state; body very open; thirst not very urgent; tongue as before. Hitherto his strength has been so little impaired that he has been able to walk alone to the night chair. Sinapisms applied to his feet, and blisters to his ancles. Observed that although these and the former blisters rose well, they occasioned so little pain, that he did not seem to feel them. 20th.—The mixture and pills were assiduously continued throughout the whole night, without creating the smallest degree of nausea; yet every thing worse. About a quarter past nine P. M. he died.

" 'I opened the body on the morning of the 21st, and as I suspected all along a morbid affection of the liver, I directed my inquiries chiefly to that viscus. Nothing could have surprised me more than the appearance it assumed. And perhaps there exists not in the annals of medicine a more curious fact than that I am going to describe. The integuments exhibited no mark of injury. On the convex side of the right lobe of the liver, I found a crucial cicatrix, one of the lines of which was in length an inch and an half, the other only an inch. At the extremity of the longest line there was a circular grangrene about two inches in diameter. On cutting open these, I found that the cicatrix penetrated about an inch into the substance of the liver, and was altogether of a

 semicartilginous semicartilaginous consistence, or something between ligament and cartilage. The grangrenous portion was at least an inch deep, and resembled rotten cork. This remarkable appearance on the liver was immediately under that part of his side on which he fell; and on more particular inquiry, I found that the corner or angle of the form or bench was that on which he fell, a circumstance which accounts for the crucial form of the cicatrix. All the other viscera were in a sound state, only the liver itself was much larger than usual.'

"I find two cases, recorded in the year 1784, of sailors, of the port of St George, Grenada, the disease in both of which was occasioned by a violent blow on the back, inflicted by tyrannical captains. One of these, by not carrying bleeding to a sufficient length, died. The other, in consequence of very copious bleeding, recovered. In both the more violent symptoms did not come on till fully a month or more after the injury was received; and in both the most prominent of these were, violent pain in the throat and breast; difficult deglutition; headache; vertigo; rigidity, and a considerable degree of insensibility of the lower extremities; obstructed respiration; and a pain stretching circularly from the pit of the stomach around the abdomen to the back, and a sensation in the region of the stomach, similar to a force drawing that part of the abdomen towards the back with great violence, and straitening of the thorax. Pulse full and quick, and surface hot and dry. In the body of the sailor who died, I found the coats of the stomach of an uncommon thickness; and all the vessels having the appearance of a preparation well injected with wax. The small intetines were sound. The great arch of the colon was considerably inflated, and its blood-vessels were turgid; and the whole of its external surface had the same appearance as that of the stomach The interior surface was either blackish, dark-brown, or dark-red, and loaded with a dark-coloured mucus. The villous coat was in most places in a dissolved state. On removing this, small hard black rough excrescences, somewhat of the nature of eschars, appeared, adhering firmly to the cellular coat. The liver was uncommonly small, and of a grey cineritious colour. It is remarkable that in these cases, the blow was inflicted in one a month, in the other two months, before any symptoms appeared, the appearance of which rendered medical aid necessary; that both patients firmly attributed their complaints to this injury; and that they had been in perfect health before the infliction of it; and that no external injury could be perceived on the part struck."

During the four years in which Dr. Chisholm has been in practice at Clifton, he has met with a few cases, the cause and early symptoms of which were similar to those of Morton and the soldier. The fatal consequences, however, which might have resulted, he thinks, were prevented by immediate bleeding to a large extent, exciting gentle ptyalism, keeping the patient tranquil, and obviating torpor of the bowels. In accidents followed by pain, uneasiness, or pyrexia, whatever organ be injured, bleeding and purging will doubtless prove beneficial; we believe, in this country the practice is universally adopted; but we cannot imagine that it will prevent fatal consequences, consequences, when the liver is actually ruptured; neither do we concur in the utility of exciting ptyalism in all such cases; what benefit could be derived from it in laceration of the spleen?

Dr. Chisholm concludes his ingenious paper with a disquisition upon the function of the spleen and a case of splenitis.

---

Art. II. *Case of Erythema Mercuriale.* By Alexander Ramsay, Assistant-Surgeon in the East India Company's Service.

The complaint which forms the subject of this communication has of late years attracted considerable notice, and has been described by different writers under various titles. Dr. Moriarty named it *Mercurial Lepra;* Dr. Spens and Dr. M'Mullins, *Erythema Mercuriale;* Mr. John Pearson, *Eczema Mercuriale;* and Sir George Alley, in a genteel quarto with elegant plates, has called it *Hydrargyria.*

The disease is very uncommon in India, and the case reported by Mr. Ramsay is the first which occurred to him, although much in the habit of prescribing mercury.

"Bechook Singh Sipahee, æt. 21, a stout healthy lad, was admitted into the hospital on the 6th April 1810, with a gonorrhœa and small chancre on the prepuce. He was ordered to use an injection of the acetate of zinc, and to take a pill containing three grains of the submuriate of mercury at bed-time. This he continued till the 12th, when he was directed to rub half a drachm of the unguent. hydrargyri into the thighs every evening. His mouth became considerably affected, in consequence of which, about the 18th the calomel was omitted. About this time also, a number of red points appeared on the inside of his thighs, which he complained of on account of the itchiness which they occasioned. These gradually increased in number, and extended over the whole thighs, genitals, and loins; appeared on the arms and sides of the chest and neck, and were accompanied with great heat. The man was naturally very black; the eruption was a dirty light red colour; did not appear to contain a fluid; was perfectly distinct, and the tops of the papulæ were completely flat. The salivation entirely ceased; his mouth and throat were swelled, and he had complained of a difficulty of swallowing for some days before. He became feverish, and had a severe headache."

The eruption was very different from any venereal one, or from any kind Mr. Ramsay had ever seen. The mercurial friction was discontinued, and the patient was ordered to take a calomel pill morning and evening.

*April 24th.* The eruption increased, was rough to the touch, and extended over most of the body except the breast. No return of salivation; face and fauces much swelled; hot and feverish; tongue foul. The calomel was increased to four four grains each dose; and he took thirty drops of laudanum at bed time.

26th. "Eruption and fever increased; pulse 120; difficulty in voiding urine; has left off the injection for some days; no salivation; belly costive. Ordered a small dose of rhubarb, with ten grains nitrate of potass. Cont. alia."

On the 30th the eruption, exudation, and fever, having increased, Mr. Ramsay began to suspect that they were occasioned by the remedy which he employed. He therefore discontinued the mercury, and gave bark. During the following month the patient suffered extremely from a *horridly* offensive discharge from most parts of the body, which, by drying on the surface, formed incrustations. The pulse kept up about 120; strength much reduced, tongue furred, restless nights; the appetite very great. His distress was increased by a large boil on the spine of the left ilium, which having broken, was succeeded by another in the right axilla.

From the 2d of June he continued to recover gradually, and by the 10th of July appeared perfectly well; but Mr. Ramsay not being certain that the venereal taint was eradicated, and wishing to ascertain whether the erythema would recur on renewing the mercurial action in the system, again put him on a course of mercury. Salivation was soon excited, and in the space of a month the eruption appeared nearly as before; the mercury was discontinued and in a few days the eruption and exudation disappeared, and the general health was good.

---

### Art. III. *Analysis of the Cactus Coccinillifer.* By John Bostock, M. D. Liverpool.

The cactus coccinillifer has been recommended as an article of nutrition, and especially as possessing antiscorbutic properties. The analysis of it was undertaken by Dr. Bostock to ascertain whether it contained citric acid.

"A portion of the leaf of the cactus, which weighed 100 grs. was cut into small pieces, and was then strongly agitated in a bottle with ℥i of water. It was then squeezed through a linen strainer, and nearly one ounce of a mucilage was procured, which was of about the consistence of albumen ovi, and was so tenacious, that it was scarcely possible to divide it into separate portions. Another ounce of water was then added to it, and it was again strongly agitated in a bottle, by which means it was reduced to a consistence similar to that of the serum of the blood, and in this state was made the subject of experiment. The residue of the leaf, that had not passed through the strainer, weighed 73 grs. but after being some time exposed to a gentle heat, it was reduced to a little more than 5 grs. The mucilage was nearly transparent transparent and colourless, and without any peculiar taste or smell. It remained for a considerable time exposed to the atmosphere, without undergoing any alteration, but gradually it deposited a small quantity of a flocculent matter, and acquired a slightly putrid odour. Although the consistence of the mucilage appeared to be very nearly the same with that which is produced by dissolving one part of gum Arabic in ten parts of water, yet it was found, upon evaporation, to contain not more than 1-100th of its weight of solid contents. There was an obvious difference between the consistence of the cactus mucilage and that from gum Arabic; a difference which, I conceive, will be comprehended, by stating that the latter is more glutinous, and the former more tenacious. In this respect the mucilage of the cactus approaches to that of linseed. The mucilage, although it appears to be indefinitely soluble in water, is not easily incorporated with it; but by strong agitation in a bottle a complete union may be formed. By the test of litmus, the mucilage was found to be very slightly acid. The quantity of acid was so small, that 250 grs. had its acid apparently neutralized by one drop of a solution of potash, formed by dissolving one part of the kali purum of the pharmacopœia in four parts of water; the addition of three drops caused the mucilage to be decidedly alkaline. This acid could not be the oxalic, because the oxalate of ammoniac threw down a minute quantity of a powdery precipitate, proving that the fluid contained lime. In order to ascertain whether this lime was united to an excess of citric acid, lime water was gradually added to convert the supercitrate into the citrate of lime, but no precipitate was produced. To try whether it was the tartaric acid which existed in the mucilage, potash was added very gradually, but no precipitate was formed. I do not, indeed, conceive that these experiments prove the absolute non-existence of either the citric or tartaric acids, because the methods employed for detecting them do not appear applicable to discover them when in very minute quantity. This, indeed, appeared to be the fact; for when very dilute solutions of the citric and tartaric acids were formed, neither the lime water in the one case, nor the potash in the other, threw down any precipitates. I conceive, however, that we may be warranted in concluding, that the power of the cactus coccinillifer does not depend upon any acid which it contains. I afterwards examined the action of some of the reagents, which I had formerly found to produce peculiar effects upon different vegetable mucilages. Although lime-water did not throw down any of the powdery precipitate which indicated the formation of the citrate of lime, yet I observed, that after lime-water had been for some time in contact with the mucilage, the sides of the glass were coated with what appeared to be small particles of gummy matter; and I found the same effect to be produced in a greater degree by barytes water. It would appear to arise from a weak action between the mucilage and the earth, by which a partial separation is produced of the vegetable matter from the water that holds it in solution. When alcohol was added to a mucilage of about the consistence of serum, that is, when it contained about 1-200th of its weight of solid matter, the two fluids were incorporated by agitation in a bottle, without any visible change being effected; but when the mucilage contained 1-100th of its weight of solid matter, the alcohol separated it in the form of a spongy spongy mass, without rendering the fluid in any degree opaque; in this respect agreeing with the mucilage of linseed, and differing from that of gum. The oxymuriate of tin, and the nitrate of silver, also formed a spongy precipitate, while the acetate of lead produced a copious dense precipitate. Silicated potash, the oxysulphate of iron, and the nitrate of mercury had no effect on the mucilage.

These experiments show, that the substance which enters so largely into the composition of the cactus coccinillifer is, in most respects, similar to the mucilage of linseed, and that, if the distinction which I formerly* pointed out between gums and mucilages is to be considered as having any foundation, the substance in question must be placed in the latter class."

(*To be continued.*)

* Nicholson's Journal, 18, 38.

